# The Double-Edged Sword—How Human Papillomaviruses Interact With Immunity in Head and Neck Cancer

**DOI:** 10.3389/fimmu.2019.00653

**Published:** 2019-04-02

**Authors:** Hao-fan Wang, Sha-sha Wang, Ya-Jie Tang, Yu Chen, Min Zheng, Ya-ling Tang, Xin-hua Liang

**Affiliations:** ^1^State Key Laboratory of Oral Diseases, Department of Oral and Maxillofacial Surgery, National Clinical Research Center for Oral Diseases, West China Hospital of Stomatology (Sichuan University), Chengdu, China; ^2^Key Laboratory of Fermentation Engineering (Ministry of Education), Hubei Key Laboratory of Industrial Microbiology, Hubei Provincial Cooperative Innovation Center of Industrial Fermentation, Hubei University of Technology, Wuhan, China; ^3^State Key Laboratory of Oral Diseases, Department of Oral Pathology, National Clinical Research Center for Oral Diseases, West China Hospital of Stomatology (Sichuan University), Chengdu, China; ^4^Department of Stomatology, Zhoushan Hospital, Wenzhou Medical University, Zhoushan, China

**Keywords:** human papilloma virus (HPV), head and neck squamous cell carcinoma (HNSCC), immune escape, immune responses, immunotherapy

## Abstract

Patients with human papilloma virus (HPV)-associated head and neck squamous cell carcinoma (HNSCC) have remarkably better prognosis, which differs from HPV-negative oropharyngeal squamous cell carcinoma (OPSCC) with respect to clinical, genomic, molecular, and immunological aspects, especially having the characteristics of high levels of immune cell infiltration and high degrees of immunosuppression. This review will summarize immune evasion mechanisms in HPV-positive HNSCC, analyze the host various immune responses to HPV and abundant numbers of infiltrating immune cell, and discuss the differences between HPV-positive HNSCC with cervical cancer. A deeper understanding of the immune landscape will help new concepts to emerge in immune-checkpoint oncology, which might be a valuable add-on to established concepts.

## Introduction

Head and neck cancers, mainly comprising squamous cell carcinoma, are the sixth commonly diagnosed cancer in the world with nearly 600,000 new cases per year (1, 2). Head and neck squamous cell carcinomas (HNSCC) represent a heterogeneous group of tumors located in the oral cavity, oropharynx, hypopharynx and larynx (3). Generally, tobacco and alcohol are two main and classical risk factors for HNSCC. Recently, human papilloma virus (HPV) has been regarded as another important pathogenic factor for HNSCC, especially for oropharyngeal squamous cell carcinoma (OPSCC). HPV 16 with a prevalence over the 80% in OPSCC is the most common among the HPV types that can cause HNSCC, followed by HPV18 (3%) (4). HPV have been thought to have specific propensity to squamous cell epithelium and take advantage of micro lesions in the surface epithelium to access the basal cell layer to initiate their infection (5). It has been reported that HPV may lead to gene alteration (6), microRNA expression (7), epigenetic changes (8), and immune system, thereby inducing the emergence of HNSCC. However, the specific and detailed molecular mechanisms of HPV contributing to HNSCC remain unclear.

To study this special immune landscape of HPV-positive HNSCC, some important differences between HPV-positive HNSCC and HPV-negative HNSCC need to be understood. Compared to HPV-negative HNSCC, HPV-positive HNSCC usually belongs to non-keratinizing, undifferentiated, or basaloid squamous cell carcinomas (9, 10). HPV-positive HNSCC has a more favorable prognosis, lower recurrence rate, and longer overall survival (OS) time because of higher sensitivity to chemotherapy and radiotherapy. The clinical differences between HPV-positive HNSCC and HPV-negative HNSCC are shown in Table 1 (9–15). HPV-positive HNSCC is primarily associated with the oropharynx, more specifically the tonsil or base of tongue where the crypts and the reticulated epithelium (16) play key roles in the immune responses. This indicated that the immune system might play an important role in HPV-associated HNSCC.

**Table 1:** Differences between HPV-positive HNSCC and HPV-negative HNSCC.

**Clinicopathologic factors**	**HPV-negative**	**HPV-positive**
Risk factors	Alcohol, tobacco	HPV, Number of oral sex partners (11)
Age	Older	Younger
Site	Anywhere	Oropharynx (base of the tongue, tonsil)
Lymph node metastases	Anyone	N2b to N3 (12)
Distant organs metastases	Lung	Unusual sites: brain, skin (13)
Stage	Anyone	stage III–IV (14)
Histopathological features	Keratinizing	non-keratinizing or basaloid (9, 10)
Tumor differentiation	Anyone	Undifferentiated (9, 10)
Outcomes	Worse survival	Better survival (12)
Sensitive to chemoradiotherapy	–	Better (15)

*Adapted from Solomon et al. (3)*.

The immune system has been shown to play a crucial role in determining whether acute or chronic HPV infection can develop into cancers, which is consistent with previous study showing that HPV infections have an increased prevalence and persistence rate in immunosuppressed individuals, such as organ-transplant recipients and human immunodeficiency virus-positive individual (17, 18). HPV and the host immune system influence mutually. HPV-positive HNSCC is recognized as a special type of cancers with features of increased levels of immune cells infiltration and high degrees of immunosuppression (19). On one hand, HPV has evolved several mechanisms to escape the recognition and clearance of host immune system. On the other hand, many studies have demonstrated that the host immune system plays an important role in the better prognosis of HPV-positive HNSCC. Therefore, this review will summarize the interaction between HPV-positive HNSCC and host immunity to provide a more detailed understanding of the immune landscape in HPV-associated HNSCC, which may be helpful to the development of promising immunotherapy.

## Immune Escape

**Figure 1:**
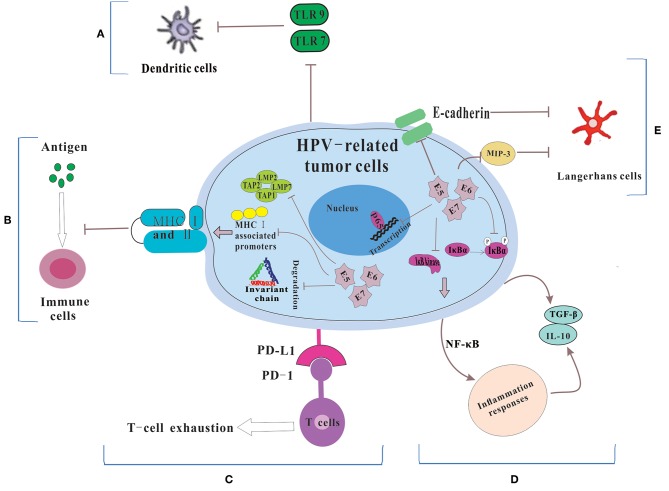
The mechanisms for HPV-related tumor cells to escape immune system. **(A)** Because of the down-regulation of TLR 7 and 9, DCs could not be activated. Thus, HPV could not be recognized by the immune system. **(B)** HPV E5, E6 and E7 cause the low expression of MHC class I and II via repressing MHC I-associated promoters, preventing the breakdown of invariant chain and inhibiting antigen processing machinery components, which lead to the failure of the process of antigen presentation. **(C)** HPV-related tumors cells express PD-L1 which interacts with PD-1 to let T cell exhausted. **(D)** HPV prevents IκB kinase activation, inhibits IκBα phosphorylation and interferes with NF-κB p65-dependent transcriptional activity to manipulate the NF-κB signaling and regulate inflammation responses. Then some immunosuppressive inflammatory mediators (e.g., IL-10 and TGF-β) are released. **(E)** HPV influences the number and activity of LCs via reducing the levels of E-cadherin and interfering with MIP-3 transcription.

Dunn et al. (20) regarded immune escape as the process that tumor cells evaded the powerful antitumor function of the immune system through various mechanisms so as to survive and proliferate in the body. HPVs are non-enveloped, small DNA viruses including nearly 8 kb circular, double-stranded DNA genome (4). HPV genome could be divided into two coding regions and one non-coding regions. The coding regions are denoted E for “early” proteins including E1, E2, E4, E5, E6, and E7 and L for “late” proteins including L1 and L2. Among them, E6 and E7 are two main oncoproteins of HPV (21, 22), which mediates cell cycle regulators p53 degradation and inactivates retinoblastoma protein (pRb), respectively. While the role of the immune system in disease progression are dual (23), the immune system still has a potent but not constant effect in clearing viral infections. Therefore, in order to survive and replicate in the hostile, antiviral environment of the host, HPV has evolved several immune evasion strategies, such as maintaining a low profile, interfering with MHC-mediated antigen presentation, suppressing T cell function, regulating the inflammatory response, and modulating antigen presenting cells (APCs) (Figure 1).

### Failure of Immune System Recognition

Maintaining a very low profile is a key strategy used by HPV to cause the failure of recognition by the immune system (24, 25). A possible explanation of low profile is that the non-lytic natural of HPV leads to the absence of pro-inflammatory signals, then the dendritic cells (DCs) are not activated and fail to move into the local environment (25). During viral infection and tumorigenesis, upregulated DNA damage response was usually accompanied, enhancing the expression of poliovirus receptor (PVP) (26), which was found to be expressed on DCs and involve in immune evasion (27). In addition, Toll-like receptor (TLR) can activate DCs, leading to increased production of pro-inflammatory cytokines, as well as increased antigen presentation (28). HVP E7 have been found to repress the transcription of the double-stranded DNA innate sensor TLR9 via inducing an inhibitory transcriptional complex consisting of NF-κBp50–p65 and ERα (29). These indicated that HPV infection could inhibit the activation of DCs through regulating the expression of TLRs. Recently, there was a study showing that TLRs 2, 3, 4, 5, 7, and 9 were expressed in OPSCC. Among them, the expressions of TLRs 5, 7, and 9 were associated with the HPV statuses of OPSCC (30). In HPV-positive HNSCC, the expression of TLR 5 and 7 was related with tumor recurrence. And high level of TLR 5 and low level of TLR 7 expression were associated with poor survival of OPSCC. The level of TLR 9 was significantly lower in HPV-positive tumors, but TLR 9 seemed to have no prognostic value (31). However, in HPV-positive HNSCC, whether HPV infection could inhibit the activation of DCs and the role of TLRs during this process remains unclear.

Other possible reasons of low profile are to keep a low copy number at the HPV infected areas and keep latent state in the host (32). In addition, because the life cycle of HPV does not have a blood-borne phase and only few replicating virus are exposed to the immune system, the host immune system is hardly able to detect the virus (25).

### Interference With Antigen Presentation

T cells including CD8+ cytotoxic T lymphocytes (CTLs) and CD4+ helper T cells (Th) play an important role in host immune system to eliminate foreign virus infections. T cells cannot effectively exert its antiviral effect without the major histocompatibility complex MHC (known as human leukocyte antigen HLA in humans), which can recognize and present antigens to T cells in immune responses. It has been shown that HPV may disrupt MHC I and II protein function and escape the recognition of CTLs, NK cells and Th cells, leading to the failure of immune surveillance. E7 protein, expressed by HPV16 and 18 was found to induce a repression in MHC class I heavy chain promoter. Besides, HPV 18 E7 resulted in the repression of a bidirectional promoter, and HPV 16 E7 could not (33). Moreover, HPV 16 E5 only down-regulates surface expression of HLA-A and HLA-B, but no down-regulation was found in the natural killer (NK) cell inhibitory ligands HLA-C and HLA-E (34). The overexpression and polymorphisms of HLA-G, a non-classical HLA class I (Ib) molecule with the capacity of suppressing the cytotoxic activity of T and NK cells, were found to be associated with HPV infection and the development of cervical cancer (35), whereas no correlation between HPV and HLA-G was observed in oral squamous cell carcinoma (OSCC) (36). HPV 16 E5 has also been shown to diminish surface MHC class II expression which acted a momentous role in delivering antigens for recognition by CD4+ T cells via preventing the breakdown of invariant chain (Ii) (37). However, the intensity of HLA II expression was unexpectedly higher in HPV+ OSCC compared with HPV- tumors (38). The increased expression of HLA II may be due to elevated production of IFN-γ mediated by CD8+ and CD4+ T cells (39).

In both HNSCC samples and cell lines, the expression of antigen processing machinery components (including proteasome subunits low molecular mass protein LMP2 and LMP7, and the transporter proteins TAP1 and TAP2) and HLA I antigen were down-regulation or even loss. And a positive correlation between patients' survival and the expression levels of these five makers was observed (40). Besides, some immune cells enriched with these 5 molecules (especially TAP2) which were functionally associated with the cross-presentation pathways had a powerful effect on the proliferation of cytotoxic CD8+ T cells (41), whose presence in the tumor microenvironment was described to be of better prognosis (as described below). Although there are still some contradictions, it could be suggested that HPV proteins-mediated down-regulation of MHC molecules will be a mechanism to protect them from immune surveillance through inhibiting antigen recognition and presentation.

### Utilization of PD-1: PD-L1 Interaction

The view that programmed death 1 (PD-1) is a key immune-checkpoint receptor expressed by activated thymocytes, mature T and B cells and myeloid cells and mediates immune evasion has been confirmed in many types of cancer, such as non-small-cell lung cancer, melanoma, or renal cell cancer (42, 43). Because of various immune cells that can express PD-1, PD-1 may have a broader role in immune regulation. Two ligands for PD-1, both belonging to the B7 family, which have been identified as PD-L1 (B7-H1) and PD-L2 (B7-DC), have the immunosuppressive effect as well. Interaction of PD-1 and PD-L1 has been described to promote T-cell exhaustion, which works for CD4+ as well as for CD8+ T cells, hence suppressing immune surveillance (43).

In HNSCC, cytotoxic CD8+ T cells expressing a persistent high level of PD-1 resulted in “T cell failure,” which could lead to an invalid immune response (44). Scognamiglio et al. found that 72% HNSCC expressed PD-L1 in both tumor cells and immune cells. In cervical lymph node of the metastatic samples, PD-L1 was also found (45). Ukpo et al. observed a positive association between PD-L1 expression and distant metastasis in both HPV-positive and HPV-negative HNSCC (46). PD-1 blockade could enhance T-cell function and possibly change the immunosuppressive phenotype in patients with HNSCC (47). A phase 1b trial showed that pembrolizumab, a PD-1 inhibitor, was a safe, well-tolerated and antitumor drug in patients with recurrent or metastatic HNSCC irrespective of HPV status (48). Importantly, HPV-positive HNSCC was found to express higher PD-L1 proteins compared to HPV-negative HNSCC (49). In the tumor microenvironment of HPV-positive HNSCC, multiple cells, including a mixture of epithelial-derived tumor cells, and recruited CD68+ tumor associated macrophages (TAMs) have the ability to express PD-L1 protein. In the tonsillar crypts where initial HPV infection occurred, PD-L1 presented membranous expression. An “immune-privileged” site was created there, allowing the tumors to gain the ability of adaptive immune resistance, probably because the PD-1: PD-L1 interaction could functionally suppress the capacity of tumor infiltrating lymphocytes (TILs) to produce effector cytokines and subsequent promote virus-induced malignant transformation (50). Oddly, although the expression of PD-L1 was negatively related with the infiltration of CD8+ T cells (51), the significant associations between the expression of PD-L1 and HNSCC patients' survival were not observed (44, 46, 51).

### Regulation of the Inflammatory Response

Epidemiological surveys proved that several types of tumors were significantly associated with pathogens that caused persistent infections, such as cervical cancer and HPV, hepatitis B viruses and hepatocellular carcinoma, Epstein-Barr virus and B-cell non-Hodgkin's lymphoma, *Helicobacter pylori* and gastric adenocarcinoma (52). The most important characteristic of these chronic infections was chronic inflammation. The functional relationship between chronic inflammation and cancer has been well-tested (53). Persistent chronic inflammation could promote tumor progression at all stages of tumor development via dysregulating specific cellular pathways, such as TLR and TGF-β pathways (54). Inflammation could play an immunosuppressive role to help tumors avoid immune surveillance (55).

There are several mechanisms for HPV to regulate the inflammatory response, including manipulating the NF-κB signaling and regulating the expression of a cascade of inflammatory cytokines (56). The E7 protein prevents IκB kinase activation and IκBα phosphorylation, thereby reducing NF-κB activity and NF-κB binding to DNA. The E6 protein interfere with NF-κB p65-dependent transcriptional activity (57).

On the other hand, HPV-regulated expression of inflammatory cytokines also can influence the inflammatory responses and then produce an immunosuppressive microenvironment. In cervical cancer, HPV up-regulated interleukin 10 (IL-10) and transforming growth factor (TGF)-β to avoid the antitumor immune responses (58). The levels of IL-10 and TGF-β were higher in HPV-positive OSCC patients than that in normal individuals (59). And E6 protein could stimulate IL-10 expression in both OSCC cells and cervical cancer cells. In HPV-positive OSCC patients, a negative association between high level of IL-10 mRNA and the 5-year survival rate was observed. The possible explanation was that up-regulation of IL-10 not only promoted tumor cell growth rate and migration capability, but also suppressed T-cell immunity, leading to a persistent HPV infection and the progression of HPV-positive OSCC (60). In addition, HPV16-positive oropharyngeal cancer patients had a higher level of TGF-β than HPV-negative patients. Increased level of TGF-β could influence immune and inflammation response to viral infection and form an immunosuppressive state, in turn increasing the susceptibility to HPV infection and promoting tumors progression (61).

### Modulation of Langerhans Cells

Langerhans cells (LCs) acting a role as APC can identify danger signals in the environment and present antigens to T cells in the context of MHC, thereby initiating the antigen-specific immune responses. In HNSCC, these cells could activate immune responses and act as APCs in the defense against tumors (62). Thus, decreased LCs density suggest reduced immune surveillance.

Three HPV related oncoproteins including E5, E6, and E7, can regulate the activity and number of LCs via different mechanisms (63). The expression of HPV E6 protein was associated with the reduced levels of E-cadherin, which medicated the adhesion between keratinocytes (KC) and LC and contributed to adequate LC deposition (64). HPV E6 and E7 proteins were found to interfere with macrophage inflammatory protein 3 (MIP-3) transcription, which leaded to a reduced migration of immature LCs and a reductive level of immune surveillance at the area of HPV infection (65). In cervical intraepithelial neoplasia (CIN), HPV could play an immunosuppressive role by decreasing the number of LCs (66). Lasisi et al. (67) showed a decreased amount of LCs in OSCC. Another reason for decreased density of LCs may be enhanced LCs migration to draining lymph nodes to present antigens (68). However, Kindt et al. (69) suggested that the level of LCs was higher in HNSCC patients, while LCs infiltration was significantly lower in HPV-positive HNSCC than in HPV-negative tumors. And increased LCs number was associated with better prognoses in HPV-negative patients, but no significant correlation was shown in HPV-positive patients. Thus, although the number and prognostic value of LCs in HNSCC were still controversial, the above data suggested that regulating LC number might be an immune escape mechanism of HPV-related lesions and cancers.

In addition, HPV E5 could damage tumor necrosis factor ligand (FasL) and tumor necrosis factor-related apoptosis-inducing ligand (TRAIL)-mediated apoptosis, thus protecting HPV-infected cells from apoptosis (70). HPV E6 and E6-associated protein could target Bak and Bax, two key pro-apoptotic factors having the canonical function of inducing apoptosis via the mitochondrial pathway (71). HPV E7 could perturb the DREAM (DP, RB-like, E2F and MuvB) complex via binding to the retinoblastoma tumor suppressor family member p130 protein to promote cellular proliferation (72). IFN-γ treatment could up-regulate the expression of antigen processing machinery components and HLA I antigen, and promote T-cell recognition in HPV-positive HNSCC (73). Therefore, the more detailed understanding of these evading mechanisms, the more efficient therapeutic strategies to improve the immune surveillance capability will be developed.

## Immune Responses

**Figure 2:**
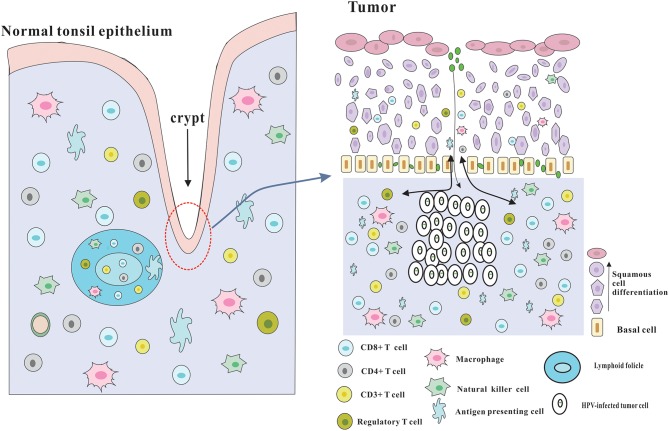
Architecture of the tonsils and possible explanation for the immune responses of HPV-positive HNSCC. The palatine and lingual tonsils are the most common infection sites for HPV. The tonsils which are lined with stratified squamous epithelium are part of oropharyngeal lymphoid tissues and have several lymphoid follicles in the lamina propria. And the epithelium invaginates into the underlying tissue to form tonsillar crypts which are covered by reticulated epithelium. These crypts are able to greatly enlarge the tonsillar surface area, thereby dramatically improving the efficiency of antigen capture and immune surveillance. The reticulated epithelium that has an incomplete basal cell layer help to the presentation of antigens and the infiltration of immune cells and APC. Besides, the permissive nature serves to virus infection without the required microlesion in cervical cancer. And HPV-related HNSCC has a significantly increased infiltration of immune cells, especially CD8+ T cells in the tumor microenvironment, which could be partly due to the special characteristics of the crypts and reticulated epithelium.

Although several immune evasion mechanisms have been mentioned above, HPV-positive HNSCC has a better prognosis, and is more sensitive to radiotherapy and chemotherapy compared with HPV-negative HNSCC, which may be due to the effective immune responses to viral and abundant numbers of infiltrating immune cells (3) (Figure 2). The role of adaptive immunity in the development of cancer is still controversial, and likely 2-fold (23). On one hand, immune cells may release inflammatory mediators with pro-tumor angiogenic and anti-apoptotic effects, which could contribute to tumor progression (74). On the other hand, adaptive immunity also plays a role of preventing tumor development, which is consistent with clinical observations that HPV-associated cancers are more prevalent in immunosuppressed patients, such as the HIV-positive patients and organ-transplant recipients. Following part will focus on the beneficial effects of adaptive immunity.

### Humoral Immunity

Adaptive immune responses can be divided into humoral immune responses and cellular immune responses. Humoral immunity mainly relies on plasma cells derived from B cells to produce antibodies to fight against foreign viruses. HPV antibodies positivity appears to be a potential prognostic and diagnostic biomarker for HNSCC. Smith et al. (75) found that 67% of HPV-positive HNSCC was E6 and/or E7 seropositive. And patients with E6 and/or E7 seropositive seemed to have a longer recurrence-free survival than the E6 and/or E7 seronegative group. Consistent with their conclusion, Lang Kuhs et al. (76) suggested that HPV16 E6 antibodies existed at the time of diagnosis in the vast majority of patients with HPV-positive OPSCC. Patients without an HPV16 E6 antibody response at diagnosis tended to have a significantly higher recurrence rate. The sensitivity and specificity of HPV16 E6 antibodies were 89.7 and 96.0%, respectively. In addition, Kreimer et al. (77) showed that HPV16 E6 seropositivity could be detected in prediagnostic plasma of patients diagnosed with OPSCC more than 10 years ago.

Another serologic marker is antibodies to HPV-derived virus-like particles (VLP), which could be a substitute of HPV infection. But it was less effective than antibodies to HPV-16 E6 or E7 oncoproteins in predicting risks of cancer. The possible explanation of this difference was that HPV VLP antibodies could be detected in healthy controls, while E6 or E7 antibodies were almost not (78). However, the gold standard methods for assigning the HPV tumor status using E6 or E7 antibodies has not been established (76). Thus, larger studies using highly specific HPV E6 or E7 serologic assays are needed.

### Cellular Immunity

#### Tumor Infiltrating Lymphocytes (TILs)

Many studies have suggested that the better prognosis of HPV-positive HNSCC was largely due to a higher number of T cells infiltrating within the tumor microenvironment, compared to HPV-negative HNSCC (79–82). There is a statistical result showing that the 3-year survival rates for patients with HPV+/TIL^high^ OPSCC, HPV+/TIL^mod^ tumors, and HPV+/TIL^low^ tumors were 94, 72, and 56%, respectively. Of note, the survival rates of individuals with HPV+/TIL^low^ tumors and patients with HPV-negative OPSCC (3-y survival rate 51%) were similar (83). Among lymphocytes infiltrating in the tumor microenvironment, CD8+ T cells, which have the capacity to produce IFN-γ and IL-17, thereby activating a strong immune response (84), are the most concerned. Many studies have suggested that increased levels of both circulating and tumor infiltrating CD8+ T cells seemed to be related with a better survival in HPV-positive HNSCC, suggesting that not only the systemic but also local immune responses play a key role in the better clinical performance of HPV-positive tumors. Wansom et al. (85) suggested that patients with percentage of circulating CD8+ T cells levels >24% tended to have an improved survival and better response to chemoradiotherapy. Besides, they also considered the CD8+ T cells levels, which were significantly related with HPV status, as a more predictive indicator than HPV-16 status alone. Solomon et al. (86) showed that abundant intratumoral and stromal tumor infiltrating CD8+ T cells were associated with improved overall survival in HPV-positive OPSCC. Hoffmann et al. (87) proposed that compared with HPV-negative OPSCC, a significantly increased frequency of E7-specific CD8+ T cells, which had the ability of releasing IFN-γ, were detected in the circulation of patients with HPV-positive tumors. Similarly, Masterson et al. (88) suggested that CD4+ and CD8+ T cells could be detected in response against HPV E6 or E7 in more than 60% of HPV-positive OPSCC patients. And elevated E7-specific CD8+ T cells were related with improved disease free survival.

In addition, Welters et al. (89) reported that the percentage of HPV-specific CD4+ T cells, which was associated with improved survival, was higher than that of CD8+ T cells in HPV-positive OPSCC. And HPV-specific CD4+ T cells could release IFN-γ and TNF-α, which had the capacity of synergizing with cisplatin-based therapy to control tumor cells growth. This could be a possible mechanism explaining the better sensitivity of HPV-positive tumors to chemotherapy and radiotherapy. By contrast, Nordfors et al. (81) found no association between the number of CD4+ T cells and overall survival of patients in HPV-positive TSCC, while high CD4+ T cells seemed to affect clinical outcome in HPV-negative tumors. Moreover, several studies proposed that a lower CD4+/CD8+ ratio had been confirmed to be obviously associated with a better overall survival in HPV-positive HNSCC (85, 90).

Furthermore, CD3+ T cells have been shown to have a positive effect on prognosis of several types of human cancer (91). Similarly, a study of 139 patients showed that a significantly increased CD3+ T cells density was found in both tumor and stromal regions of HPV-positive OPSCC, which was associated with better survival. And patients with high stromal infiltration of CD3+ CD8+ T cells had the best clinical outcome (80). However, Kong et al. (92) proposed that the number of CD3+ T cells was directly correlated with HPV status in HNSCC. But the density of CD3+ T cells was associated with significantly better survival only in the HPV negative or HPV weak positive group, while CD3+ T cells seemed to have no obvious prognostic impacts on HPV-positive tumors.

In sharp contrast with the role of PD-1 on T-cell exhaustion, Badoual et al. (93) suggested that an increased infiltration of PD-1 expressing T cells, including PD-1+CD4+ and PD-1+CD8+ T cells, significantly correlated with a favorable clinical outcome of patients with HPV-positive HNSCC. And a hypothesis to explain the contradictory results was that a part of PD-1+ T cells in the tumor microenvironment are not exhausted because of the lack of co-expression of Tim-3 and PD-1 in these cells. Besides, PD-1+ T cells expressed the higher levels of HLA-DR and CD38, which may repress tumor growth. Similarly, Solomon et al. (86) showed that HPV-positive OPSCC with high levels of PD-L1 expressing intratumoral immune cells had a significantly better overall survival. However, they did not find a relation between PD-L1 expression on tumor cells and prognosis. They also suggested another function of PD-L1+ intratumoral immune cells was to synergize with PD-1/PD-L1 checkpoint inhibitors and predict the response to PD-1/PD-L1 blockade therapy.

#### Regulatory T Cells (Tregs)

Tregs could be divided into naturally occurring regulated T cells (n T-regs), which predominantly comprise CD4+CD25+ Tregs and induced adaptive regulatory T cells (a T-regs or iT-regs) which mainly release two kinds of immunosuppressive cytokines, TGF-β and IL-10. FoxP3 is a relatively specific marker and a key regulatory gene for Tregs, especially for CD4+CD25+ Tregs (94). Tregs activity could impair effector T cell responses and promote viral infection, such as hepatitis C virus (HCV) and mycobacterium tuberculosis (Mtb). The effects of Tregs on immune effector cells may promote immune escape to lead to carcinogenesis. And the infiltration of Tregs has been shown to be closely associated with the prognosis for certain tumors (95).

CD4+CD25+ Tregs not only had increased in number in the circulation and tumor tissues of patients with HNSCC (96), but also became more immunosuppressive due to expressing more TGF-β-associated the latency-associated peptide (LAP) and the glycoprotein A repetitions predominant (GARP) as well as ATP-hydrolyzing CD39 on their surface in the blood of HNSCC patients treated with chemoradiotherapy. These dangerous Tregs leaded to suppression of antitumor immune responses and development or recurrence of HNSCC (97).

Surprisingly, Tregs seem to have a completely distinct role in HPV-assoicated HNSCC. Lukesova et al. (98) suggested that higher infiltration of Tregs and lower ratio of CD8/Tregs had a positive impact on the survival of HPV-positive HNSCC. Their explanation for the different roles of Tregs were that Tregs may not only impair Th17-cell-dependent proinflammatory and tumor-enhancing response, but also maintain HPV-positive status in some HNSCC tumors. Wansom et al. (99) proposed that increased FoxP3+ Tregs infiltration, which correlated directly with CD4+ and CD8+ infiltration, was significantly associated with lower T stage and improved survival in both HPV-positive and HPV-negative OPSCC. Furthermore, Nasman et al. (100) investigated the association between the levels of Tregs and HPV status. A significantly higher level of FoxP3+ Tregs in HPV-positive TSCC was found compared to HPV-negative tumors. A higher CD8+/Foxp3+ T cell ratio significantly correlated with a better survival in patients with HPV-positive TSCC. Thus, the role of Tregs in the prognosis of HNSCC is still controversial and the interplay between Tregs and HPV is still unclear. Much need be further explored.

#### Natural Killer (NK) Cells

In general, NK cells are considered to be components of innate immune defense. NK cells can induce the death of several target cells, including virus-infected cells and tumor cells, in the absence of antibody. Recently, NK cells have been shown to contribute to adaptive immunity as well. NK cell–mediated killing of target cells and cytokine production, such as IFN-γ, could promote host T and B cell immune responses (101). Impaired NK cells or NK cell deficiency were found to be associated not only with virus infections, such as HPV, but also with an increased incidence of various types of cancer, including HPV-related cancers (102). Consistent with this, Wagner et al. (103) found that the abundance of CD56+ cells, which mainly represented cytotoxic NK cells, were significantly higher in HPV-positive OSCC compared to HPV-negative tumors, which seemed to be responsible for the favorable outcome in HPV-positive OSCC.

#### Tumor Associated Macrophages (TAMs)

TAMs are a class of immune cells present in high numbers in the microenvironment of solid tumors. TAMs have a stark different function compared to macrophages derived from healthy or inflamed tissues, possibly because of their exposure to tumor-derived molecules, such as IL-4, IL-10, and TGF-β (104). Macrophages are generally classified into two subtypes according to participation in particular immune responses. M1 macrophages activated by IFN-γ are involved in the responses of type I helper T cells (Th1) which have a pro-inflammatory and cytotoxic (anti-tumoral) function, whereas M2 macrophages undergoing “alternative” activation by IL-4 and IL-10 are involved in Th2-type responses which are anti-inflammatory (pro-tumoral) (105, 106). In OSCC, an increased level of M2 macrophage in the tumor microenvironment was observed, which could contribute to local and systemic immunosuppression via TGF-β production (107). However, M1 macrophages had a markedly increased infiltration in HPV-positive HNSCC while the levels of M2 macrophages were similar in HPV-positive and HPV–negative HNSCC. Thus, the ratio of M1/ M2 was significantly higher in HPV-positive tumors, which was associated with a favorable prognosis (108). This implies that HPV influences the distribution of M1 and M2 macrophages in the tumor microenvironment of HNSCC, while the exact mechanisms are still unclear.

Except for an efficient immune response to foreign virus, especially a high level of CD8+ T cells in the tumor microenvironment, there are some other reasons contributing to the better prognosis and astonishing sensitivity to radiotherapy and chemotherapy of HPV-positive HNSCC, including lower expression of epidermal growth factor receptor (EGFR), overexpression of p16, a decreased p53 mutation rate and lower hypoxia (109, 110). However, whether and how HPV influences some immune cells and immune cells regulate relative genes, such as EGFR and p53, remains unclear. So much need be done to address the role of the host immune system in HPV-positive HNSCC.

## Comparison With Cervical Cancer

Despite the same etiological factor of cervical cancer (2) and HNSCC, the composition and function of their immune cells infiltration are substantially different. Only 32% patients with cervical cancer were able to detect HPV-specific T cells (111), whereas 71% patients of HNSCC had HPV-specific T cells present locally (112). Besides, approximate half of the patients with cervical cancer could not detect HPV16-specific CD4+ T cells responses. The other half had a severely impaired immune response with a defect in inflammatory cytokines production, such as IFN-γ and IL-5 (113). And the differences in CD4+ T cells infiltration could be associated with the differences in survival of HPV-positive OPSCC and cervical cancer patients (114). In contrast with the role of Tregs in HPV-positive HNSCC, increased Tregs infiltration was significantly associated with worse clinical outcome in cervical cancer patients (115). These different immune responses may be partly due to the different anatomical sites, the special tissue architectures including the crypts and reticulated epithelium, and the high load of co-infections in the oropharynx (112). In addition to immune responses, HPV-positive HNSCC and cervical cancer have differences in terms of chromosomal alterations (116), patterns of gene expression (117), and miRNA profiles (118).

## Conclusions

HPV, as a virus with the carcinogenic potential, has evolved several mechanisms to escape immune surveillance to persist and promote the development of HNSCC, though the concrete mechanisms are still obscure. While HPV could divide HNSCC into two clinically, genomically, molecularly, and immunologically distinct subgroups, the current treatments for HPV-positive and HPV-negative HNSCC are still similar. Surgical resection remains the primary treatment, and radiotherapy and chemotherapy are used as the auxiliary way, which are associated with substantial morbidity and toxicity. Immune evasion have been shown to play a vital role in the progression of HPV-positive HNSCC, while the efficient immune responses play a positive role in the favorable clinical performance of HPV-positive HNSCC. Thus, immunotherapy has been considered as a more promising treatment. And some progresses have been made in immunotherapy.

Two prophylactic vaccines, Gardasil® and Cervarix®, have been approved for use by the U.S. Food and Drug Administration (FDA). Both of them protect against HPV types 16 and 18, and have received licensure for use for the prevention of cervical cancers. However, whether they have the impact on preventing the occurrence of HNSCC has not been well-studied (119).

In addition, Nivolumab and Pembrolizumab, PD-1 inhibitors, have got FDA approval for their indication for the treatment of patients with recurrent and/or metastatic HNSCC with disease progression on or after platinum-containing chemotherapy (120). Other treatments, such as DNA vaccines (121), E6/E7peptide vaccines (122), virus-like particle (VLP)-based vaccines (123), and recombinant vaccines (124), albeit still on trial, also provide attractive prospects for the development of immunotherapy. Developing efficient immunotherapies for clinical treatment that can not only help to inhibit the repression role but also enhance the promotion of the immune system requires a more detailed understanding of the immune microenvironment of HNSCC. Therefore, more efforts are needed to explore regulatory mechanisms of the immune system.

## Author Contributions

All authors listed have made a substantial, direct and intellectual contribution to the work, and approved it for publication.

### Conflict of Interest Statement

The authors declare that the research was conducted in the absence of any commercial or financial relationships that could be construed as a potential conflict of interest.
